# Strategies for Improving Transdermal Administration: New Approaches to Controlled Drug Release

**DOI:** 10.3390/pharmaceutics15041183

**Published:** 2023-04-07

**Authors:** Olimpia Dumitriu Buzia, Ana Maria Păduraru, Claudia Simona Stefan, Monica Dinu, Dorin Ioan Cocoș, Lawrence Chukwudi Nwabudike, Alin Laurențiu Tatu

**Affiliations:** 1 Centre in the Medical-Pharmaceutical Field, Faculty of Medicine and Pharmacy, “Dunarea de Jos” University of Galați, 800008 Galați, Romania; 2 N. Paulescu Institute, 030167 Bucharest, Romania; 3 Clinical Medical Department, Faculty of Medicine and Pharmacy, “Dunarea de Jos” University, 800008 Galati, Romania; 4 Dermatology Department, “Sf. Cuvioasa Parascheva” Clinical Hospital of Infectious Diseases, 800179 Galati, Romania; 5 Multidisciplinary Integrative Center for Dermatologic Interface Research MIC-DIR, 800010 Galati, Romania

**Keywords:** transdermal patches, iontophoresis, sonophoresis, electroporation, microns

## Abstract

In this work, we aim to address several strategies to improve transdermal drug delivery, such as iontophoresis, sonophoresis, electroporation and micron. We also propose a review of some transdermal patches and their applications in medicine. TDDs (transdermal patches with delayed active substances) are multilayered pharmaceutical preparations that may contain one or more active substances, of which, systemic absorption is achieved through intact skin. The paper also presents new approaches to the controlled release of drugs: niosomes, microemulsions, transfersomes, ethosomes, but also hybrid approaches nanoemulsions and microns. The novelty of this review lies in the presentation of strategies to improve the transdermal administration of drugs, combined with their applications in medicine, in light of pharmaceutical technological developments.

## 1. Introduction

A transdermal delivery system is a painless method of drug administration through intact skin [1].

Transdermal patches favor the controlled release of active ingredients through the skin into systemic circulation. Drugs administered through such systems escape first-pass metabolism, and the steady state is maintained, similar to a continuous intravenous infusion, for up to several days [2]. Controlled release forms of drugs, such as microcapsules, nanoparticles, liposomes, chitosan and vancomycin microspheres and encapsulated biopolymers, amongst others [3,4,5,6,7,8], have begun to occupy an important place in therapy due to the many advantages they possess, in terms of the release of active substances, slowly, over a long period, via a single administration, which is very convenient for the patient. For example, in rheumatism, there are a multitude of preparations on the market, but through the development of drugs with controlled release, the opportunity has been created for the realization of these transdermal pharmaceutical forms having piperine and capsaicin [9] as active substances. with beneficial results for the patient [10].

Since 1980, impressive growth has been seen in this area, with much commercial success [10].

**Figure 1:**
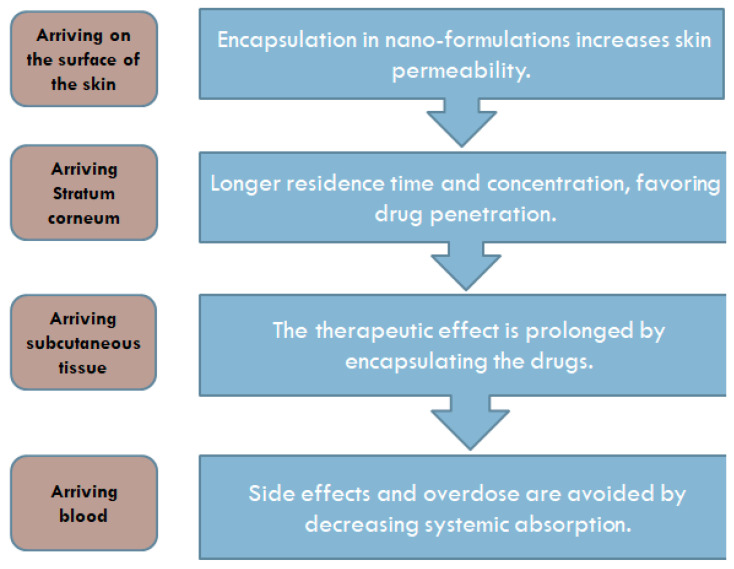
Highlights of the benefits of drug delivery using nano-formulations [13].

This progress made has been based on a clearer understanding of skin barrier function and the physicochemical, pharmacokinetic and physiological factors that support the feasibility of transdermal administration [11]. One of the important fields of nanotechnology is nano-formulations [11]. Bearing in mind their reduced particle size, nanoformulations have a better effect on drug retention, specificity and targeting (Figure 1) [12], making them an ideal TDDS [12].

Nano-formulations can be divided into vesicles including liposomes, transfersomes, ethosomes, niosomes, invasives and nanoparticles including lipid nanoparticles, polymeric nanoparticles, (Table 1) as well as nano-emulsions [13].

Each drug molecule has unique physicochemical properties, which are decisive to achieving an efficient transdermal delivery system [13].

## 2. Brief History

The first transdermal therapeutic systems (TTSs) were aimed toward the treatment of angina pectoris using nitroglycerin, and examples include:

- Transderm NitroR—Alza, Ciba Geigy;
- NitrodurR—Kay Pharmaceuticals.
- Some of the first transdermal patches with a medicinal substance that were launched on the pharmaceutical market were those used for motion sickness (USA—1981; France—1986) [13,14].

They were originally designed to combat the nausea astronauts suffer, but then, they were used for any kind of motion sickness (on cars, ships, planes, etc.) [13]. Currently, commercial applications of iontophoresis include the intradermal administration of lidocaine as a local anesthetic and dexamethasone for local inflammation [13,14]. The devices used are usually plasters connected to a power supply via cables [14].

Transdermal therapeutic systems have been used in the treatment of Alzheimer’s disease, Parkinson’s, ADHD, depression [15], and to help individuals quit nicotine, and they do not cause systemic reactions, which can occur with the use of other drug administration systems [16].

During a meeting of the American Society of Plastic Surgeons [14], the news of the appearance of ActiPatch TM therapy was received with joy. It incorporates a microchip that can deliver continuous therapy for many weeks to relieve pain and speed up wound and scar healing [14,15]. The existence of a microchip that can act as a continuous source of energy is interesting in future research on iontophoresis [13,14,15].

## 3. Strategies to Improve the Administration of Drugs in the Form of Transdermal Patches

Substances with analgesic effects can be administered on the skin in the form of topical patches made to produce local effects or transdermal patches that ensure the controlled and prolonged release of the active substance [17,18,19,20,21].

Currently, numerous local analgesics and anesthetics (nonsteroidal anti-inflammatory drugs, opioids, capsaicin, lidocaine, and tetracaine) are formulated as patches [17,21].

Transdermal patches are indicated for use in cases of severe pain due to their systemic effects. In this way, the opioids fentanyl and buprenorphine are administered [17,21].

Fentanyl was the first medicinal substance used in the treatment of pain in the pharmaceutical form of a transdermal patch, it has an analgesic effect that persists for 72 h [22,23].

Buprenorphine is an analgesic that has been used in pharmaceutical practice for a long time, but with the appearance in the pharmaceutical form of a transdermal patch with simple application and long-lasting effects (the administration of one patch every 7 days), patient compliance has increased [24,25,26]. It is also used in substitution therapy for drug-dependent patients [27].

Neuropathic pain is a chronic and complex painful condition which can be debilitating and difficult to treat. Nerve fibers can be damaged, dysfunctional or destroyed [27].

The calcitonin-gene-binding peptide (GCRP) has a very important role in chronic neuropathic pain and is a neuropeptide that is released from sensory endings [28]. The local anesthetics currently used tend to produce the non-specific blocking of motor and sensory functions [28].

### 3.1. Energy-Based Methods

At the beginning of the 20th century, the concept of ion therapy was introduced, and it was demonstrated that “ionic drugs” can penetrate the skin and exert important local and systemic effects [29].

Iontophoresis is a technique that uses low-intensity electric currents to increase the absorption of drugs through the skin [29].

**Figure 2:**
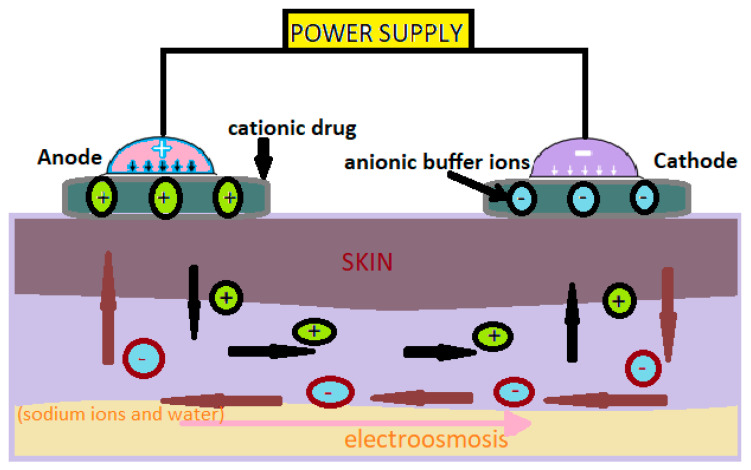
Administration of cationic drugs via iontophoresis. Mechanism [30].

The values of the intensity of the electric current used in iontophoresis are between 0.5 and 20mA (Figure 2) [29].

This technology is successfully used by dermatologists to treat patients with hyperhidrosis [29].

The method uses an electrical device to inject a low-voltage current into the affected area. It is painless and relatively safe, but its effectiveness is sometimes similar to a topical antiperspirant [29].

Iontophoresis is mainly used to increase skin absorption of ionizing drugs, but neutral or weakly charged molecules can also be administered through electroosmosis [30].

Iontophoresis has been investigated with the administration of various types of drugs, including nonsteroidal anti-inflammatory drugs (ibuprofen, aspirin, and indomethacin) [29], granisetron, donepezil, and insulin [30].

In this technology, neutral or cationic products are positioned under the anode and anionic products under the cathode [31]. The low-intensity electric current used moves the ions through the skin. This method can be used to administer medicinal substances for local or systemic effects [31].

In ophthalmology, atropine, scopolamine, gentamicin and fluorescein are being tested [31]. In dentistry, they are used as local anesthesia in cases of multiple dental extractions [31,32]. The administration of systemic drugs (fentanyl [31], certain insulin formulas, antihypertensives, antirheumatics, antidiabetics, hormones, etc.) via iontophoresis is still under investigation [32].

**Figure 3:**
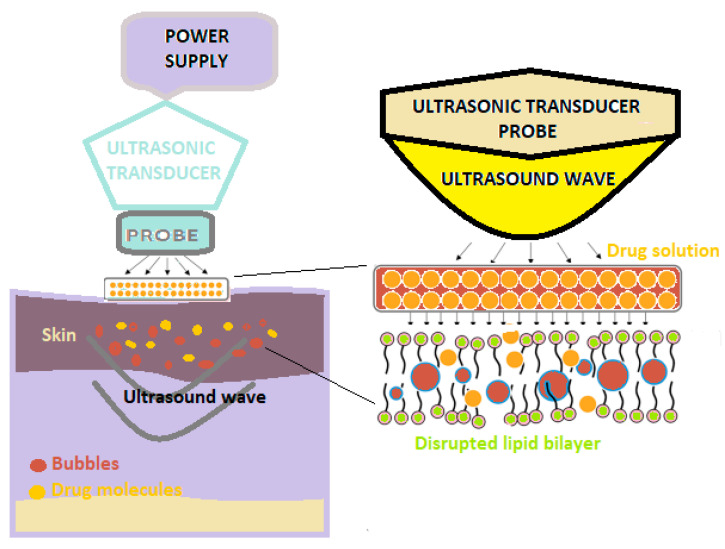
A schematic illustration of sonophoresis-assisted transdermal drug delivery [30].

Sonophoresis is a method of increasing the absorption of drugs through the skin, which uses ultrasounds with frequencies varying between 20 kHz and 16 MHz to modify the structure of the lipid layer of the stratum corneum (Figure 3) [31].

The use of ultrasound results in an increase in skin temperature and, implicitly, the better absorption of the medicine at this level [30].

In this technique, a predetermined frequency of ultrasound is used on the drug solution that is positioned under the probe, as well as on the skin [31,32].

Various studies have shown that ultrasound-mediated transdermal delivery had good results in the case of the administration of some proteins (erythropoietin, interferon y and insulin), ketoprofen [33] and vancomycin, which demonstrates that a wide range of hydrophilic and hydrophobic products, as well as macromolecules and small molecules, can be administered through sonophoresis [30,31,32].

The result of the sequential ultrasound procedure is so-called sonophoresis. Sonophoresis is a process of the penetration and migration (spreading) of dermo-cosmetic substances in the skin under the action of these sequential ultrasounds [30].

**Figure 4:**
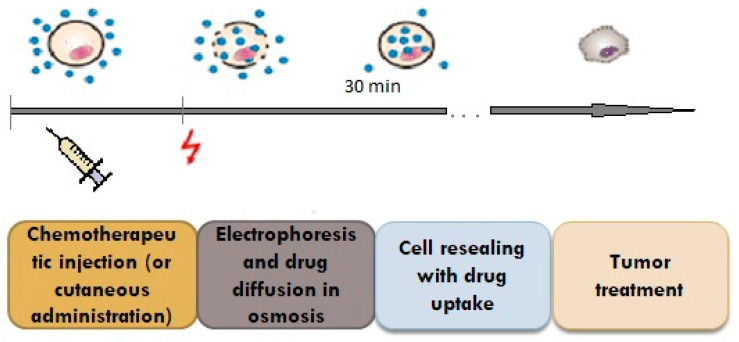
Penetration of the drug through the pores created by the impulse.

Electroporation is a method used to facilitate the transdermal delivery of drugs; it is based on the penetration of the skin and the creation of micropores by using high voltages (between 10 and 1000 V) for a duration of less than a few hundred milliseconds (Figure 4) [30,31].

**Figure 5:**
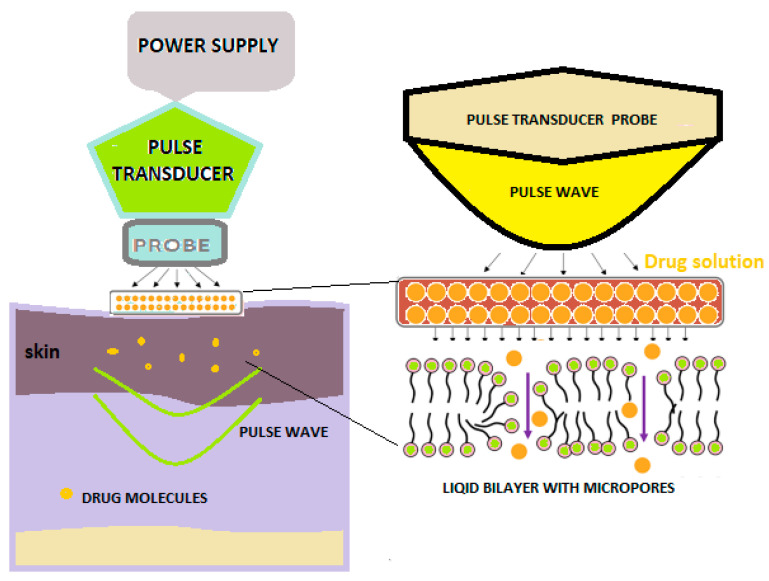
Schematic diagram showing pulse wave transdermal drug delivery [30].

With the help of pulse waves, aqueous pores are created in the double lipid layer of the stratum corneum, the solution with the active substance on the skin penetrates deeply, and thus, superior absorption is achieved (Figure 5) [30].

By using this method, good results have been reported regarding the in vitro absorption of insulin, tetracaine, thymol and fentanyl [31].

Blagus et al. performed in vivo research using this method on rats, following the degree of absorption of fentanyl, dextrin and doxorubicin [32].

Galvanization achieves superior absorption in the skin [30].

**Figure 6:**
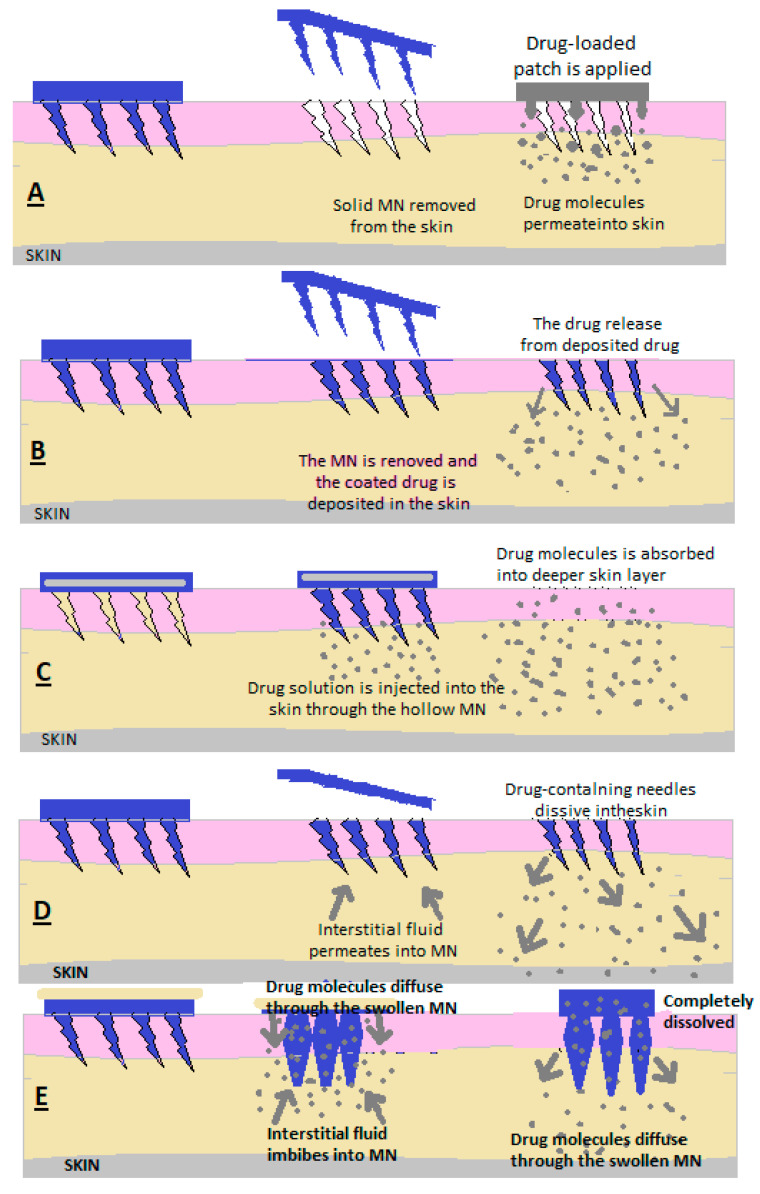
Drug release through (**A**) solid MN, (**B**) covered MN, (**C**) empty MN, (**D**) dissolving MN and (**E**) MN that forms a hydrogel [30].

Microns (MNs) are defined as needles with heights between 25 μm and 2000 μm, and the materials used in their preparation determine their applications [30,31]. The principle of the microneedle release system consists of the creation of microns that penetrate the stratum corneum, which limits absorption, to create micropipes through which the medicine is deposited in the dermis. (Figure 6) [30].

This new technology presents multiple advantages from many points of view:

- MNs can penetrate the stratum corneum, avoiding contact with nerves and capillaries, offering a faster, efficient, painless and comfortable delivery compared to other transdermal administration methods [30,31,32,33].
- It does not require specialized personnel for administration.
- By dissolving and forming the hydrogel, MNs eliminate the need to use specific measures for the disposal of needles and syringes, and the risk of contamination or accidental reuse is avoided [34,35,36].
- The patient’s skin can regenerate very quickly after the removal of MNs; thus, there is only a very low probability of infection and irritation at the application site, as well as possible occurrences of isomorphic lesions due to the Koebner phenomenon, such as lichen planus and psoriasis [34,35,36].

### 3.2. Solid MNs

MNs are used to make micropipes with an absorption role in the patient’s skin; after removing the needles, a transdermal patch is applied with a medicinal substance that is released through passive diffusion in the skin [35,36].

### 3.3. Covered MNs (Figure 6)

Covered MNs are obtained through different methods to ensure uniform coverage [35,36]. A coating is used consisting of the medicinal substance combined with surfactants and thickening agents, which ensure adhesion to the needle ends [35,36]. The needle tips are coated with a formulation in the form of a solution or dispersion [34,35,36]. To make the MNs, polymers or metals are used [34,35,36].

After the MNs have penetrated the skin, at the moment of contact between the interstitial liquid and the coating, it is dissolved and absorbed [34,35,36].

### 3.4. Empty MNs

Empty MNs are formulated to release a drug continuously into the skin through needle holes [30,31,32,33]. The solution or dispersion containing the medicinal substance is loaded inside the MNs and then administered transdermally [30,31,32,33]. (Figure 6c) Empty MNs and solid MNs can be made of glass, polymers, silicon and metals [30,31,32,33].

As an advantage, compared to solid MNs and coated MNs, MNgol has a greater capacity to fill with medicine [30,34].

### 3.5. MN of Dissolution

Following application to the skin, the needles also dissolve from the MN matrix, and the active substance is released slowly (Figure 6d) [30].

In the case of the manufacture of these forms of transdermal administration methods, mixtures of biocompatible and soluble polymers with medicinal substances are used [30].

Polymers can be vinyl alcohol, polycarboxymethylcellulose, vinylpyrrolidone, hyaluronic acid and copolymers of methyl vinyl ether and maleic acid [30].

### 3.6. MN Hydrogel Former

A reservoir containing the active substance is embedded in the empty MN upon application, and after penetration through the skin, the MN swells due to the absorption of interstitial liquid, the reservoir dissolves and the medicine is absorbed through the already-dilated ducts [30,33]. A hydrogel mass is formed to control the release of the drug depending on the crosslinking power of the hydrogel network. This allows the drug to be released slowly over a long period (Figure 6e) [30,33].

In dentistry, special needle-based devices could be very useful and beneficial, because oral carcinoma is often oral squamous cell cancer and is very difficult to detect in the early stages, being asymptomatic [30,33]. These special devices can detect elevated levels of biomarkers, such as Cyfra 21-1, TPA, CA-125 antigen, MMP-9 and TNF-α [37,38].

Microneedles can be used in dental periodontal surgery as an adjuvant because they can accelerate the healing process and locally increase platelet-derived growth factor (PDGF), transforming beta and alpha (TGF-β and TGF-α), connective growth factor and fibroblasts (FGF) [38,39]. They can potentiate neocollagenases and neovascularization through intercellular matrix layering and fibroblast proliferation [39].

Antimicrobial patches that hold gingival-grip microneedles could help speed up the healing of periodontal lesions [39,40,41]. These microneedles are loaded with antimicrobial agents, possibly with green tea and nano-silver, and these patches have been used in experiments designed to suppress the microbial load at the periodontal wound site [40,41].

Following a study, rat experiments demonstrated that analgesic microneedle (AMN) patches have been developed using pain-free soluble content microneedles with the transdermal transfer of the selective antagonist peptide CGRP to treat localized neuropathic pain [42].

Compared to conventional therapies, AMN patches produced a satisfactory analgesic effect without influencing the motor function of laboratory animals, indicating the high specificity of the peptides released against CGRP receptors [42].

### 3.7. Hybrid Approaches

Recently, the use of a combination of a stainless steel nanoemulsion and microns was studied to improve the transdermal delivery of aceclofenac [43].

### 3.8. Transdermal System with Amorphous Content of Flurbiprofen and Lidocaine

Researchers created an amorphous molecular complex by combining different anti-inflammatory substances (flurbiprofen, etodolac, naproxen, piroxicam and indomethacin) and lidocaine and examined the physicochemical properties of the complex [44,45,46,47].

The focus was on intermolecular flurbiprofen–lidocaine interaction. They found that the solubility of flurbiprofen was 100 times higher than the product alone, and the solubility of lidocaine was 2 times higher [46]. The conclusion was that it was possible to develop a transdermal preparation with anti-inflammatory and analgesic effects useful for treating the pain of various diseases [46,47].

### 3.9. Transdermal System with Flurbiprofen and Lidocaine—Ionic Liquids

Ionic liquids (ILs) are ionic salts in a liquid state over a wide temperature range (between ambient temperature and below 100 °C), with high polarity and very high ionic conductivity, high thermal stability, negligible vapor pressure, low volatility and viscosity, non-flammability and a very good recycling capacity [47,48,49].

Third-generation ionic liquids comprise an innovative class of functional materials with improved properties, characterized by excellent biological activity, due to their physical–chemical and biological properties, low toxicity and especially their biotechnological and pharmaceutical potential [50,51,52,53]. These ILs are distinguished by surprising antimicrobial and antibacterial, antibiofilm (bacterial resistance) and antitumor pharmaceutical activity [50,51,52,53].

The role of third-generation biodegradable ILs has been highlighted in the case of transdermal drug delivery systems [54]. Within these systems, ILs can play various roles: as active pharmaceutical ingredients, in drug modification or as transdermal permeation enhancers, as individual agents or in combination with other agents or as chemical permeation enhancers [54].

The incorporation of active pharmaceutical ingredients (APIs) brings about efficient API-IL drug delivery systems, which currently represent an innovative solution for improving the pharmacokinetic and pharmacodynamic properties of many less water-soluble drugs by increasing their solubility, bioavailability, permeability and stability, as well as by eliminating polymorphisms [47,55].

IL molecules are able to act as ideal solvents (green solvents) for drug substances, allowing topical and transdermal administration [56]. Incorporating ILs into water, oils or hydroalcoholic solutions bring about topical IL-based drug administration systems, which have very good therapeutic efficiency at the level of the hydrolipidic film of the skin barrier, due to the increase in drug solubility and the increase in the local permeability of the skin [47,56].

Cholinium-amino-acid-based ionic liquids display high efficiency in the transdermal delivery of ferulic acid and puerarin by improving the solubility and permeability of these two APIs [56].

The skin release capacity of hyaluronic acid has been improved using different choline-based ionic liquids, the strongest being obtained for an IL consisting of choline and citric acid [57].

1-butyl-3-methylimidazolium hexafluorophosphate and 1-hexyl-3-methylimidazolium chloride showed 5% higher antimicrobial activity and higher skin penetration levels [58].

In vitro studies on the production of insulin biopolymer films based on biocompatible ILs have also been performed to improve the transdermal permeability of insulin [59].

A study showed that ionic fluids are safe, contribute to the increase in skin permeability and are promising applications in the field of transdermal systems used in pain treatment [60].

The design of non-irritating drug-IL systems, with high efficiency for permeability through the skin, is based on the effects of the structure of counterions: polarizability, their molecular weight and their polar surface area [61].

The therapeutic potential of the topical delivery of siRNA (small interfering RNA) using ionic liquids (ILs) for the treatment of genetic skin diseases is enhanced by the use of choline-geranic acid ionic liquid [62].

### 3.10. Capsaicin–Diclofenac Transdermal System

The exact mechanism of this anti-inflammatory combination with low-dose capsaicin for the treatment of osteoarthritis pain is still under investigation [63].

### 3.11. The Potential of Bee Venom in the Treatment of Pain via Topical or Transdermal Administration

The efficacy of transdermal administration for biologics is limited due to the barrier function of the stratum corneum and possible adverse reactions [64].

Bee venom has been studied since the early 19th century and has been shown to have a complex composition that confers numerous potential therapeutic uses [65,66,67,68].

Many studies are conducted specifically to test the anti-inflammatory effect of bee venom and its mechanism of action [69,70,71,72,73].

Efforts are being made to discover safe practices combined with modern delivery systems to minimize adverse reactions [74].

Melittin in the composition of bee venom is responsible for the anti-inflammatory action and is therefore the most studied compound [75,76,77,78,79,80].

### 3.12. Micromelitin

A study was conducted in rodents by administering melittin transdermally with microns [81].

The substance was loaded into hyaluronic acid microneedles and significantly inhibited the progression of rheumatoid arthritis [82].

After modifying hyaluronic acid with methacrylate groups, microneedles with sustained release properties were obtained [82].

The study concluded that polymeric microns for the transdermal delivery of biologics could be a promising treatment for rheumatoid arthritis in terms of patient compliance and the therapeutic effect [82].

### 3.13. Transdermals Patch with Medicinal Plants Used in the Treatment of Pain

In China, a study was conducted to develop a transdermal patch containing *Siegesbeckiae herba* extract for the alternative treatment of rheumatoid arthritis [83].

*Siegesbeckiae herba* is an annual medicinal plant that, according to its traditional uses, chemical constituents and clinical studies, exhibits analgesic and anti-inflammatory activities and properties [84,85,86,87].

An extract prepared from *Siegesbeckia pubescens* Makino with 48.5% (*w*/*w*) kirenol was used in the preparation of transdermal patches, and its anti-inflammatory and analgesic activity was evaluated in in vitro studies [84,85,86,87].

The research results are promising; the transdermal patches showed good analgesic and anti-inflammatory effects, demonstrating potential to be used as an alternative therapy in rheumatoid arthritis [84,85,86,87].

## 4. New Approaches Regarding the Controlled Release of Drugs

Recent approaches and new formulation technologies include niosome microemulsions, liposomes, nanoemulsions, ethosomes, niozomios, etc. [27,88,89,90,91,92,93].

### 4.1. Nanoemulsions

Nanoemulsions are isotropic dispersions of oil and water, used as nanopores to improve the transdermal delivery of substances [27].

They can be incorporated into patches, facilitating penetration and supporting the release of the active substance [27]. The following anti-inflammatory substances have been studied: ibuprofen [88], aceclofenac [89], meloxicam [90], celecoxib [91], ketoprofen [92] and indomethacin [93].

### 4.2. Nanocrystal

The use of nanocrystals as vehicles for transdermal systems is of great interest [94]. Khan et al. demonstrated an improvement in the anti-inflammatory effect of capsaicin when it was incorporated as a nanocrystalline formulation compared to the marketed product [94].

Research has also been conducted in this regard on ibuprofen and flurbiprofen [27,94].

### 4.3. Transfersomes

Transfersomes are often used by researchers as substances with anti-inflammatory effects [27,95]. Duangit and Colab found that meloxicam-loaded transfer zones could be used as transdermal therapeutic systems [95].

### 4.4. Ethosomes

The active substance successfully released using ethosomes is indomethacin [96].

### 4.5. Niosomous

Surfactant-based nanovesicles have been shown to improve the transdermal release of capsaicin, used as an antirheumatic or against shingles [97,98,99].

### 4.6. Nanoparticles with Venom

The interest of researchers in finding solutions for the safe and therapeutic transdermal administration of bee venom has led to the use of the product embedded in nanoparticles as a delivery vehicle [100]. The safety of administration is still limited [101].

Nanoparticles are effective for bioactive compounds, and the venom can be transported and delivered safely, improving patient compliance [101]. Nanoparticle degradation can be controlled by changing the type and amount of polymer and molecular weight [102].

### 4.7. Transdermal Mechanism of Nano-Formulations

The mechanisms by which nanoformulations interact with the skin are different; some can be completely absorbed by the skin, others not [102,103].

Lipid-based nanoformulations, due to their structural similarity to those comprising the stratum corneum (SC), interact with the skin and have the possibility to attach to it, increasing the level of hydration [103]. This process can lead to an exchange of lipids in the intercellular lipid domain, to a change in polarity and gradually to a weak structure [102,103].

Therefore, it is important to know the mechanism of penetration of nanoformulations through the skin very well, which will lead to the creation of superior transdermal products in terms of skin penetration and absorption [102,103,104].

## 5. Conclusions

Transdermal drug management has become a research topic of interest in pharmaceutical technology, and transdermal drugs are one of the most developed pharmaceutical products on the global market.

The use of these systems may overcome the disadvantages associated with other delivery routes such as oral and parenteral.

The use of transdermal patches can circumvent/eliminate many problems associated with oral drug administration, such as first-pass hepatic metabolism, enzymatic digestion attack, drug hydrolysis and degradation in acidic media, drug fluctuations, degradation and metabolism to gut microbiota and gastrointestinal irritation.

These pharmaceutical forms allow for the maintenance of a plasma concentration of the medicinal substance between the level of therapeutic action and toxicity for long periods. This avoids complications that might have occurred if parenteral administration had been used to ensure a high blood concentration.

Micron’s technological platforms have demonstrated superiority in terms of flexibility compared to other transdermal systems through certain aspects related to the various possibilities of the intradermal administration/biotherapy of a wide range of drugs and through therapeutic monitoring. Currently, a diverse range of transdermal patches are used as cosmetic, transdermal and topical systems. Thanks to the research carried out since ancient times on the skin, the manufacturing technologies, the clinical observations and the expertise presented, the making of these patches has developed continuously, and a level of their formulation which would have been unimaginable a few years ago has been reached. Further studies are being carried out for the realization of combinations of active delivery systems with patches and systems with measured doses.

## Figures and Tables

**Table 1:** Summary of typical components, transdermal mechanisms and applications of nano-formulations [13].

Nano-Formulation Types	Nano-Formulations—Typical Components	Main Transdermal Mechanism
Liposomes	Cholesterol, phospholipid	SC (stratum corneum) liquids have a much higher compatibility with liposomes due to the effects of phospholipids; thus, skin hydration is produced by increasing the moisture of the cuticle.
Transfersomes	Edge, phospholipid, activator, cholesterol	Due to the edge activator, transferomes can undergo a higher degree of deformation from the hair follicles.
Ethosomes	Alcohol, water, phospholipid	The deformability of ethosomes and the level of solubility of drugs in the lipid state are increased by alcohols.
Niosomes	Cholesterol, phospholipid, non-ionic surfactant	Increases SC (stratum corneum) moisturizing, weakened tight cell structure; the thermodynamic activity gradient increases, and so does the drug at the interface.
Invasomes	Alcohol, phospholipid, terpene	Terpenes act as amplifiers producing penetration with the effect of disrupting the lipid sealing of the SC (stratum corneum) and increasing the transepidermal osmotic concentration through the hair follicles.
Solid lipid nanoparticles	Solid lipid, (co)-surfactant	They favor the penetration of the active substance through occlusive effects on the skin surfaces.
Nanostructured lipid carriers	solid lipid, oil (co)-surfactant	They favor the penetration of the active substance through occlusive effects on the skin surfaces.
Polymericnanoparticles	Polymer	They facilitate the production of the concentration gradients assembled on the surface of the hair follicle or the skin and then capture them like a drug reservoir, and surface charges affect the permeability, following the path of the hair follicle.
Nanoemulsions	Oil, water, (co)-surfactant	In the SC (stratum corneum), there is a disruption in the lipid arrangement and an increase in the concentration of the skin permeability gradient inside and outside.
